# 2643. Seasonality of infant respiratory syncytial virus associated bronchiolitis around the US since the onset of COVID-19: Results from four US health systems 2016-2023

**DOI:** 10.1093/ofid/ofad500.2255

**Published:** 2023-11-27

**Authors:** Adam Blatt, Mina Suh, Zachary Wolf, Emory Waddell, Christopher B Nelson

**Affiliations:** Duke University, Durham, North Carolina; Epidstrategies, Mission Viejo, California; Clinetic, Durham, North Carolina; Clinetic, Durham, North Carolina; Sanofi, Swiftwater, Pennsylvania

## Abstract

**Background:**

Respiratory syncytial virus (RSV) bronchiolitis (Br) is the leading cause of US infant hospitalizations and lower respiratory tract infections [1]. The historical seasonality of RSV in the US across all ages using passive laboratory surveillance has been described by the CDC (2014-17) [2] [3] and more recently by Staadegaard et al [4]. The objective of this analysis is to describe local variation in infant RSV-Br seasonality since the onset of COVID-19 in March 2020, across all facilities and all settings in four health systems across the US, and compare to trends from the recent pre-COVID-19 era.

**Methods:**

From October 2015-April 2023 we conducted real-time surveillance across all facilities and all settings in the State University of New York (SUNY) Upstate Medical University Health System in Syracuse, NY; the Duke University Health System (DUHS) in Durham, NC; the University of South Florida Health and Tampa General Hospital (TGH) in Tampa, FL; and the Renown Regional Medical Center Health System (Renown) in Reno, NV. We assessed electronic health records (EHR) of infants (0-11 months) using ICD-10 codes for Br and RSV-Br and laboratory testing data to describe infant RSV-Br encounters. For additional methods see Movva N et al [5].

**Results:**

During the recent pre-COVID-19 era, there was regional variation in seasonal onset and peak disease burden of infant RSV-Br (**Figure 1**). Onset and peak disease was earliest at TGH in the southeast and latest at Renown in the west. While occurring outside the historical seasonality timeframe, interseasonal 2021 and early seasonal 2022 infant RSV-Br was similar in duration and followed the same geographic pattern of onset to historical disease.

**Figure:**
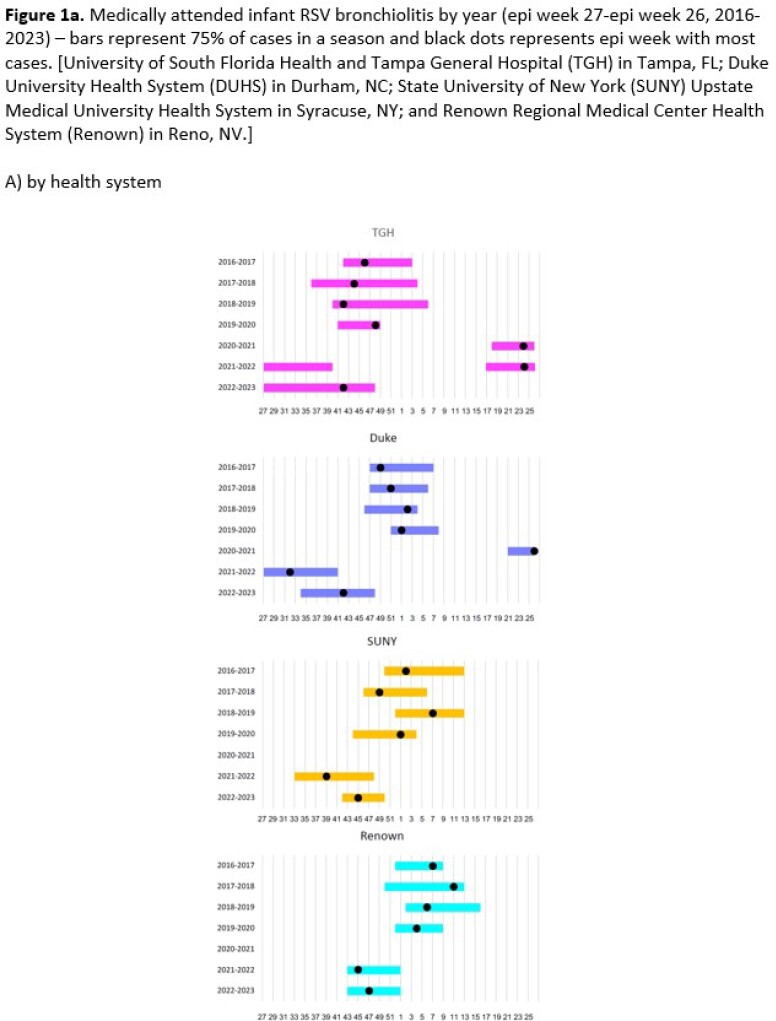

**Figure:**
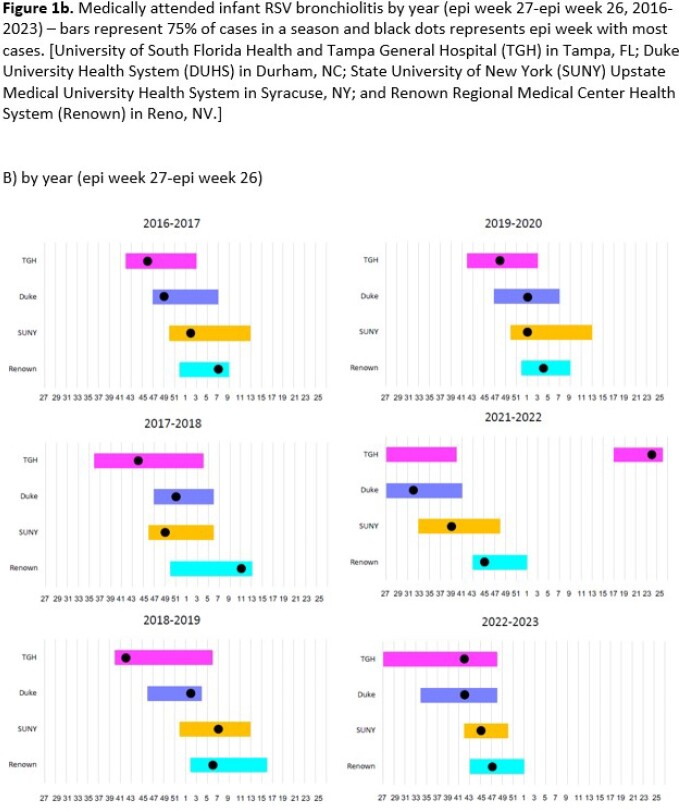

**Conclusion:**

After the US onset of COVID-19 in March 2020, the geographic pattern of RSV-Br onset and peaks was preserved, despite the shift to interseasonal and early seasonal disease in 2021 and 2022, respectively. RSV surveillance is useful to determine whether RSV seasonality is returning to typical historic patterns. These results highlight the need for health system-level surveillance and can guide discussion regarding administration of candidate RSV vaccines and immunoprophylaxis and assessment of their impact.

**Disclosures:**

**Mina Suh, MPH, International Health**, AstraZeneca: Grant/Research Support|Sanofi: Grant/Research Support|Sobi: Grant/Research Support **ZACHARY Wolf, MBA, MS**, AstraZeneca: Grant/Research Support|Sanofi: Grant/Research Support **Emory Waddell, n/a**, Sanofi: Research support **Christopher B. Nelson, PhD MPH**, Sanofi: employee|Sanofi: employee|Sanofi: Stocks/Bonds|Sanofi: Stocks/Bonds

